# Associations Between Serum Soluble α-Klotho and the Prevalence of Specific Cardiovascular Disease

**DOI:** 10.3389/fcvm.2022.899307

**Published:** 2022-06-20

**Authors:** Jun-Peng Xu, Rui-Xiang Zeng, Mu-Hua He, Shan-Shan Lin, Li-Heng Guo, Min-Zhou Zhang

**Affiliations:** ^1^The Second Clinical College of Guangzhou University of Chinese Medicine, Guangzhou, China; ^2^Guangzhou University of Chinese Medicine, Guangzhou, China; ^3^Department of Critical Care Medicine, Guangdong Provincial Hospital of Chinese Medicine, Guangzhou, China; ^4^School of Biosciences and Biopharmaceutics, Guangdong Pharmaceutical University, Guangzhou, China

**Keywords:** aging, biomarker, Klotho, cardiovascular disease, National Health and Nutrition Examination Survey (NHANES)

## Abstract

**Objective:**

Accumulating experimental evidence has identified the beneficial effects of the anti-aging protein, serum soluble α-Klotho, on longevity, and the cardiovascular system. Although a previous study has revealed the predictive value of α-Klotho on total cardiovascular disease (CVD), the associations between α-Klotho and specific CVDs, including congestive heart failure (CHF), coronary heart disease (CHD), myocardial infarction (MI), and stroke, remains to be fully elucidated in humans.

**Methods:**

For 8,615 adults in the 2007 to 2016 National Health and Nutrition Examination Survey, stratified multivariable logistic regression models, restricted cubic spline curves, and subgroup analyses were used to evaluate the associations between α-Klotho and the four specific CVDs.

**Results:**

In the quartile analyses, compared to those in the highest quartile, participants in the lowest level of α-Klotho were significantly associated with CHF [odds ratio (*OR*) = 1.46, 95% *CI*: 1.09–1.97] and MI (1.33, 1.02–1.74), which was not the case for CHD (1.12, 0.91–1.38) or stroke (0.96, 0.73–1.25). Each unit increment in the ln-transformed α-Klotho concentrations was only positively associated with a 38 and 24% reduction in the prevalence of CHF and MI, respectively. Restricted cubic spline curves indicated that the α-Klotho was correlated with CHF and MI in linear-inverse relationships.

**Conclusion:**

The present findings suggested that the serum soluble α-Klotho is significantly associated with the prevalence of CHF and MI. To better determine whether α-Klotho is a specific biomarker of CVD, particularly for CHD and stroke, further research in humans is needed.

## Introduction

Cardiovascular disease (CVD), which is broadly defined as congestive heart failure (CHF), coronary heart disease (CHD), myocardial infarction (MI), and stroke, is the leading cause of mortality and disability-related diseases worldwide (1). To date, despite the growing availability of high-quality preventive and therapeutic measures for CVD, there is considerable room for further improvement as patients with CVD distinctly have higher risks of death and recurrent hospitalization than the common healthy population. Biomarkers are good for risk stratification and prognosis evaluation of patients with CVD. Currently, left ventricular ejection fraction and B-type natriuretic peptides are regarded as generally accepted markers indicative of sensitivity to clinical therapy for CHF (2). Cardiac troponins and C-reactive protein are meaningful predictors for patients with CHD and MI. However, age-related arterial diseases are also present in the absence of conventional CVD risk factors and relevant biomarker elevation (3). Furthermore, epidemiological reports have indicated that the prevalence of CVD among Americans increases progressively with advancing age, from ~5.5% in people below 45 years of age to ~41% in people over 65 (4). Thus, CVD may be regarded as an intrinsically aging disease.

Several candidate predictors, such as telomere length, epigenetic clocks, and DNA methylation-based biomarkers, hold promise for measuring biological age (5, 6). Similarly, the anti-aging protein, serum soluble α-Klotho, encoded by the *KL* gene, has been recently highlighted as it plays a role in regulating oxidative stress and aging *in vivo* (7, 8). The α-Klotho predominantly exists in two forms as follows: a single-pass transmembrane protein and a soluble protein that circulates in the blood, urine, and cerebrospinal fluid (7). Although the α-Klotho is mainly expressed in kidneys, its expression in both human and mouse arteries has also been demonstrated (9, 10). Many studies have revealed that transgenic overexpression of α-Klotho not only extends lifespan by 20–30% (11) but also promotes cardiovascular protection, including improvement of endothelial dysfunction, hypotensive effects, alleviating cardiac fibrosis, and arterial stiffening (12–15). Increasing experimental evidence supports that α-Klotho expression is closely related to longevity and cardiovascular benefits, but investigations in humans are still limited. One previous small-scale study first found a predictive value between α-Klotho and total CVD in 2011 (16). Nonetheless, it is currently unknown whether lower circulating levels of α-Klotho may be a biomarker of specific CVDs as different subtypes of CVDs may have varied underlying pathophysiological mechanisms. However, a recent study has determined that the α-Klotho is prospectively associated with CVD mortality among the American population (17). Therefore, we initially hypothesized that the α-Klotho may also be correlated with the prevalence of specific CVDs, including CHF, CHD, MI, and stroke.

## Materials and Methods

### Study Population

The National Health and Nutrition Examination Survey (NHANES) is a series of national surveys to evaluate the health status of US citizens with a complex, stratified, multistage, and probability sampling method. The Centre for Disease Control and Prevention ratified the study protocols, and details about the surveys are available at www.cdc.gov/nchs/nhanes.

**Figure 1:**
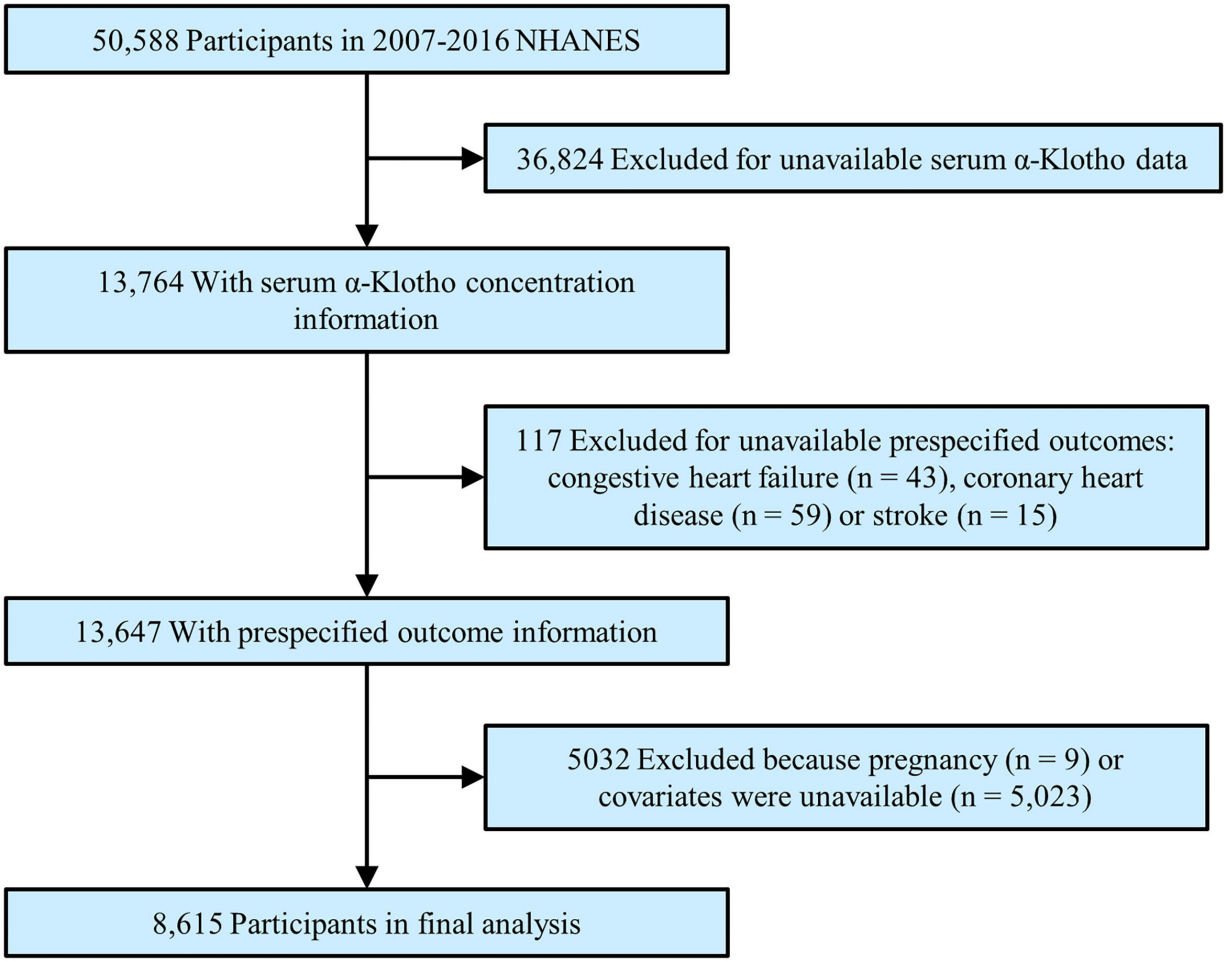
Study flowchart. Flow chart showing the process of participant selection. Of the 50,588 participants in the 2007 to 2016 National Health and Nutrition Examination Survey (NHANES), 8,615 remained in the final analysis.

To maximize the sample size, we abstracted the data into this analysis from the 2007 to 2016 NHANES. During this time, a total of 50,588 participants were initially enrolled. After excluding participants with missing α-Klotho (*n* = 36,824), who had unavailable CVD outcomes (*n* = 117), who were pregnant (*n* = 9) or who lacked covariates (*n* = 5,023), a total of 8,615 participants were enrolled in the final analysis (Figure 1).

### Serum α-Klotho Concentrations

As reported by the NHANES, serum specimens from participants aged 40–79 years old were collected, flash-frozen, and stored at −80°C at the Centre for Disease Control and Prevention. During the period of 2019–2020, the Northwest Lipid Metabolism and Diabetes Research Laboratories of Washington University received the samples and quantified individual α-Klotho concentrations using a commercially available ELISA kit (IBL International, Gunma, Japan) (18). In the laboratories, each α-Klotho concentration from humans and two quality standard samples with high and low concentrations were calculated as a mean value from duplicate sample analyses according to the manufacturer's protocol. Two standard samples with very high and high Klotho concentrations were used at different dilutions to evaluate the assay linearity and demonstrated excellent linearity in the assay measurement range (*R*^2^ = 0.998 and 0.997, respectively). The laboratories calculated the minimum detection limit to be 4.33 pg/ml, while the manufacturer reported that it was 6.15 pg/ml. The mean intra-assay and inter-assay coefficients of variation were 3.6 and 3.2% for the standard samples and 2.8% and 3.6% for the human samples, respectively. All the results were automatically transmitted to the laboratory Oracle Management System and were checked by the laboratories.

### Definition of Outcomes

In the present study, the outcomes originated from any self-reported diagnosis in the CVD-related questionnaires, including CHF, CHD, MI, stroke, and angina pectoris. All participants with CHD, MI, or angina pectoris were included in the CHD group of our research.

### Covariate Information

The covariates of this study were age, sex, race, body mass index, education, smoking, drinking, hypertension, diabetes, white blood cell counts, neutrophil-to-lymphocyte ratio, platelet counts, total cholesterol, triglycerides, low-density lipoprotein cholesterol (LDL-C), high-density lipoprotein cholesterol (HDL-C), serum calcium, serum phosphorus, urine albumin, estimated glomerular filtration rate (eGFR), glycated hemoglobin (HbA1c), total fat intake, energy intake, physical activity, and medications (such as statins, diuretics, β-blockers, antihypertension drugs, and hypoglycemic agents). Antihypertensive drugs included angiotensin-converting enzyme inhibitors, angiotensin-II receptor blockers, and calcium channel blockers. Hypoglycemic agents included oral drugs and insulin injections. The volume of individual physical activity was computed by multiplying the frequency and duration (minutes) of moderate or vigorous recreational physical activity per week. According to the latest ESC and WHO guidelines that recommend at least 150 min per week of moderate and/or 75 min of vigorous physical activity for the general population (19, 20), we derived two groups to evaluate whether the participants engaged in sufficient physical activity. The kidney function of each participant was estimated by eGFR using the Chronic Kidney Disease Epidemiology Collaboration equation (21), and chronic kidney disease in this study was defined as eGFR <60 ml/min/1.73 m^2^. The detailed acquisition process and measuring method of the remaining variables are available at www.cdc.gov/nchs/nhanes.

### Statistical Analysis

Baseline characteristics of all participants were stratified by α-Klotho quartiles with the continuous and categorical variables reported as the median (interquartile ranges) and numbers with percentages, respectively. To detect the differences across quartile groups, χ^2^ tests or Kruskal–Wallis tests were performed, in which appropriate box plots were used to examine the differences between α-Klotho concentrations and baseline categorical variables. Moreover, bivariate associations between α-Klotho concentrations and baseline continuous variables were examined using the Spearman correlation analysis.

In analyses examining associations with CHF, CHD, MI, and stroke, α-Klotho concentrations were treated as an independent variable or scaled ln-transformed, or they were divided into quartiles, using stratified multivariable logistic regression models with different adjustments to estimate odds ratios (ORs) and corresponding 95% confidence intervals (*CIs*). Model I was not adjusted for any confounders; and Model II was adjusted for age, sex, and race. Model III was fully adjusted for age, sex, race, body mass index, education, smoking, drinking, diabetes, hypertension, physical activity, white blood cell counts, neutrophil-to-lymphocyte ratio, platelet counts, total cholesterol, triglycerides, LDL-C, HDL-C, serum calcium, serum phosphorus, urine albumin, eGFR, HbA1c, energy intake, total fat intake, and medications. Model IV was only adjusted for significant difference factors at baseline.

Additionally, we generated restricted cubic spline curves with knots at the 5^th^, 35^th^, 65^th^, and 95^th^ percentiles of α-Klotho concentrations to explore the predictability and dose–response effects of α-Klotho concentrations as a continuous variable. Last, several subgroup analyses were performed by logistic regression analysis with full adjustment. First, we analyzed different subgroups, including age, sex, race, hypertension, diabetes, chronic kidney disease, smoking, drinking, and physical activity, to identify potential effect modifiers. Second, to test for statistical significance of interactions, interaction terms between different quartiles of α-Klotho concentrations and different subgroups were generated and examined by the Wald test for categorical variables. If necessary, the possible interactions between all adjusted factors were also tested. A *p-value* of < 0.05 (two-sided) was considered statistically significant. All analyses were performed with the STATA Statistical Software (Release 12.0. College Station, TX, USA), except for the restricted cubic spine curves, which were calculated by the *ggplot2* and *rms* packages in R (version 4.1.2).

## Results

### Baseline Characteristics

**Figure 2:**
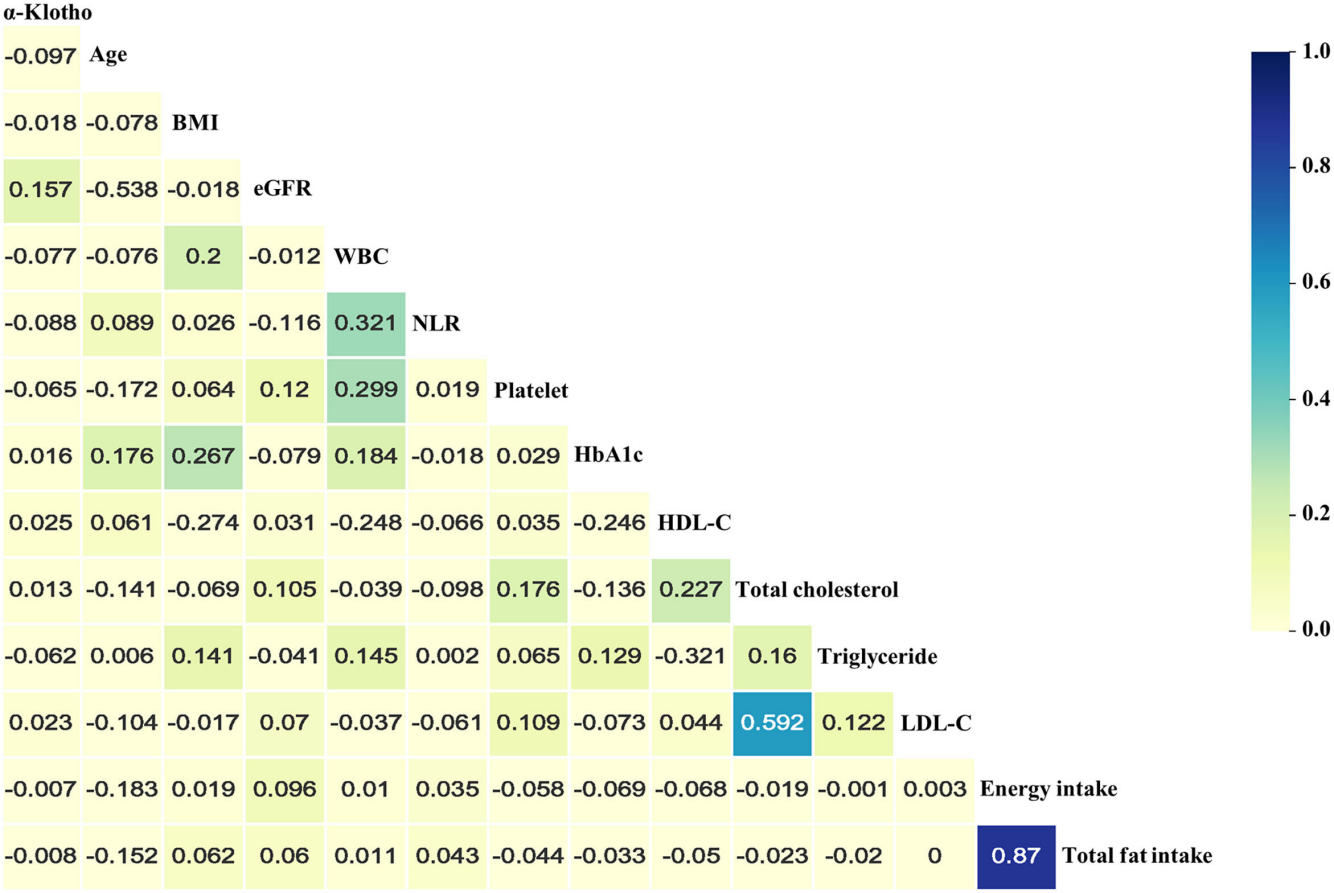
Heatmap of the correlations between serum soluble α-Klotho and baseline continuous variables using Spearman correlation analysis. Absolute *value* of “*r*” over 0.02 was significantly correlated. BMI, body mass index; eGFR, estimated glomerular filtration rate; WBC, white blood cell; NLR, neutrophil-to-lymphocyte ratio; HbA1c, glycated hemoglobin; HDL-C, high-density lipoprotein cholesterol; TC, total cholesterol; LDL-C, low-density lipoprotein cholesterol.

Table 1 presents the baseline characteristics of a total of 8,615 participants who enrolled in the study. Among all participants, 46% were male, and 48.8% were non-Hispanic Whites. The median age of participants at enrolment was 61 years old. In the present study, the median α-Klotho concentration was 797.0 pg/ml (interquartile range: 646.5–988.1 pg/ml). The proportions of CHF, CHD, MI, and stroke were 5.4, 12.1, 7.0, and 5.8%, respectively. Overall, compared to those in the highest quartile, there were more subjects in the lowest α-Klotho group who were older, male, smokers, and drinkers with worse kidney function and higher levels of urine albumin and inflammation markers, such as neutrophil-to-lymphocyte ratio, white blood cell counts, and platelet counts. Additionally, the patients in the lowest α-Klotho group had more comorbidities, including hypertension and CVD, which may account for the increased medication prescriptions. As shown in Figure 2 and Supplementary Table 1, the results of Spearman correlation analysis suggested that age, white blood cell counts, neutrophil-to-lymphocyte ratio, platelet counts, and triglycerides were weakly negatively correlated with α-Klotho concentrations but that eGFR and HDL-C were weakly positively correlated with α-Klotho concentrations. Higher α-Klotho concentrations were observed in individuals who were female, non-Hispanic Blacks, non-smokers, non-drinkers, and CVD-free. In addition, the α-Klotho concentrations did not differ by physical activity level (Supplementary Figure 1; Supplementary Table 2).

**Table 1:** Baseline characteristics of all patients according to serum α-Klotho quartile^a^.

**Characteristics**	**Total participants** **(*n* = 8,615)**	**Serum α-Klotho quartile (pg/ml)**				
		**Quartile 1 (*n* = 2,154)**	**Quartile 2 (*n* = 2,153)**	**Quartile 3 (*n* = 2,154)**	**Quartile 4 (*n* = 2,154)**	***P*** **-value**
α-Klotho, pg/ml	797.0 (646.5–988.1)	550.4 (481.6–601.3)^**^	720.9 (684.2–758.2)^**^	879.5 (835.8–930.3)^**^	1172.8 (1065.8–1343.9)	<0.001
Age, years	61.0 (52.0–69.0)	62.0 (53.0–70.0)^**^	61.0 (52.0–69.0)^**^	60.0 (51.0–68.0)	60.0 (50.0–67.0)	<0.001
Male, %	3,967 (46.0)	1078 (50.0)^**^	1048 (48.7)^**^	953 (44.2)^*^	888 (41.2)	<0.001
Body mass index, kg/m^2^	29.4 (25.7–33.9)	29.5 (26.1–33.9)	29.4 (25.7–33.8)	29.3 (25.6–34.0)	29.4 (25.6–34.0)	0.467
**Ethnicity, %**
Non-hispanic white	4,207 (48.8)	1068 (49.6)^**^	1157 (53.7)^**^	1087 (50.5)^**^	895 (41.6)	<0.001
Non-hispanic black	1,753 (20.3)	451 (20.9)^**^	365 (17.0)^**^	369 (17.1)^**^	568 (26.4)	
Other	2,655 (30.8)	635 (29.5)^**^	631 (29.3)^**^	698 (32.4)^**^	691 (32.1)	
Education, %						0.048
Lower than high school	2,266 (26.3)	586 (27.2)^**^	560 (26.0)	550 (25.5)	570 (26.5)	
High school	1,948 (22.6)	528 (24.5)^**^	474 (22.0)	496 (23.0)	450 (20.9)	
More than high school	4,401 (51.1)	1,040 (48.3)^**^	1,119 (52.0)	1,108 (51.4)	1,134 (52.6)	
Smoking status, %						<0.001
Never smoker	4,232 (49.1)	954 (44.3)^**^	985 (45.8)^**^	1,088 (50.5)^*^	1,205 (55.9)	
Current smoker	1,549 (18.0)	435 (20.2)^**^	415 (19.3)^**^	372 (17.3)^*^	327 (15.2)	
Ex-smoker	2,834 (32.9)	765 (35.5)^**^	753 (35.0)^**^	694 (32.2)^*^	622 (28.9)	
White blood cell counts, 10^3^/μl	6.9 (5.7–8.4)	7.1 (5.8–8.6)^**^	7.0 (5.9–8.4)^**^	6.9 (5.6–8.3)^*^	6.7 (5.5–8.1)	<0.001
Neutrophil-to-lymphocyte ratio	2.0 (1.5–2.7)	2.1 (1.5–2.8)^**^	2.0 (1.5–2.7)^**^	2.0 (1.5–2.6)^**^	1.9 (1.4–2.5)	<0.001
Platelet counts, 10^3^/μl	234.0 (198.0–279.0)	239.0 (202.0–283.0)^**^	237.0 (201.0–280.0)^**^	233.0 (197.0–278.0)^*^	229.5 (192.0–273.0)	<0.001
Total cholesterol, mmol/L	5.0 (4.3–5.7)	4.9 (4.2–5.8)	5.0 (4.3–5.7)	5.0 (4.3–5.7)	5.0 (4.3–5.7)	0.225
Triglyceride, mmol/L	1.3 (1.3–1.3)	1.3 (1.3–1.3)^**^	1.3 (1.3–1.3)^**^	1.3 (1.3–1.3)^**^	1.3 (1.2–1.3)	<0.001
LDL-C, mmol/L	2.9 (2.9–2.9)	2.9 (2.9–2.9)	2.9 (2.9–2.9)	2.9 (2.9–2.9)	2.9 (2.9–2.9)	0.123
HDL-C, mmol/L	1.3 (1.1–1.6)	1.4 (0.4) 1.3 (1.1–1.6)	1.4 (0.4) 1.3 (1.1–1.6)	1.4 (0.4) 1.3 (1.1–1.6)	1.4 (0.4) 1.3 (1.1–1.6)	0.143
Serum calcium, mmol/L	2.4 (2.3–2.4)	2.4 (2.3–2.4)^*^	2.4 (2.3–2.4)	2.4 (2.3–2.4)	2.4 (2.3–2.4)	0.028
Serum phosphorus, mmol/L	1.2 (1.1–1.3)	1.2 (1.1–1.3)	1.2 (1.1–1.3)	1.2 (1.1–1.3)	1.2 (1.1–1.3)	0.188
Urine albumin, mg/L	8.4 (4.3–18.8)	8.5 (4.4–23.1)^**^	8.4 (4.3–18.9)	7.9 (4.2–16.8)	8.4 (4.2–18.5)	0.004
Estimated glomerular filtration	85.6 (70.1–98.1)	80.7 (63.2–94.5)^**^	84.8 (69.7–98.0)^**^	87.0 (72.1–98.3)^**^	89.1 (75.6–100.6)	<0.001
rate, ml/min/1.73m^2^					
HbA1c	5.7 (5.4–6.3)	5.8 (5.4–6.3)	5.7 (5.4–6.2)^**^	5.7 (5.4–6.2)^**^	5.8 (5.4–6.4)	<0.001
Energy intake, kcal	1814.0 (1346.0–2392.0)	1831.0 (1339.2–2417.0)	1824.0 (1362.0–2386.0)	1801.0 (1338.2–2386.5)	1812.0 (1352.5–2376.0)	0.929
Total fat intake, gm	67.7 (44.8–97.2)	67.5 (44.0–99.5)	68.1 (45.1–97.4)	67.0 (44.5–97.3)	67.8 (45.8–94.5)	0.810
Current drinking, %	1,866 (21.7)	538 (25.0)^**^	521 (24.2)^**^	431 (20.0)^*^	376 (17.5)	<0.001
Physical activity, %						0.003
Does not meet guidelines	6,404 (74.3)	1,638 (76.0)	1,540 (71.5)^**^	1,597 (74.1)	1,629 (75.6)	
Meets guidelines	2,211 (25.7)	516 (24.0)	613 (28.5)^**^	557 (25.9)	525 (24.4)	
Diabetes, %	2,090 (24.3)	574 (26.6)	490 (22.8)^*^	480 (22.3)^**^	546 (25.3)	0.002
Hypertension, %	5,204 (60.4)	1,390 (64.5)^**^	1,257 (58.4)	1,254 (58.2)	1,303 (60.5)	<0.001
Statins, %	3,311 (38.4)	926 (43.0)^**^	866 (40.2)^**^	802 (37.2)^**^	717 (33.3)	<0.001
Diuretics, %	2,265 (26.3)	634 (29.4)	524 (24.3)^**^	501 (23.3)^**^	606 (28.1)	<0.001
β-blockers, %	1,537 (17.8)	455 (21.1)^**^	366 (17.0)	358 (16.6)	358 (16.6)	<0.001
Antihypertension drugs, %	4,152 (48.2)	1,136 (52.7)^**^	1,034 (48.0)	988 (45.9)	994 (46.1)	<0.001
Hypoglycaemic agents, %	2,001 (23.2)	551 (25.6)	469 (21.8)	471 (21.9)	510 (23.7)	0.009
**Outcomes, %**
Congestive heart failure	461 (5.4)	165 (7.7)^**^	111 (5.2)	96 (4.5)	89 (4.1)	<0.001
Coronary heart disease	1,046 (12.1)	318 (14.8)^**^	259 (12.0)	250 (11.6)	219 (10.2)	<0.001
Myocardial infarction	599 (7.0)	198 (9.2)^**^	153 (7.1)^**^	137 (6.4)	111 (5.2)	<0.001
Angina pectoris	378 (4.4)	102 (4.7)	88 (4.1)	93 (4.3)	95 (4.4)	0.771
Stroke	499 (5.8)	153 (7.1)^*^	117 (5.4)	113 (5.2)	116 (5.4)	0.028

*LDL-C, low-density lipoprotein cholesterol; HDL-C, high-density lipoprotein cholesterol; HbA1c, glycated hemoglobin; Antihypertensive drugs included angiotensin-converting enzyme inhibitors, angiotensin-II receptor blockers, and calcium channel blockers; Hypoglycaemic agents included oral drugs and insulin injection*.

a *Values for categorical and continuous variables are expressed as n (%) and median (interquartile ranges), respectively*.

* *p < 0.05 and*

** *p < 0.01, compared to quartile 4*.

### Associations of α-Klotho With Specific CVDs

**Figure 3:**
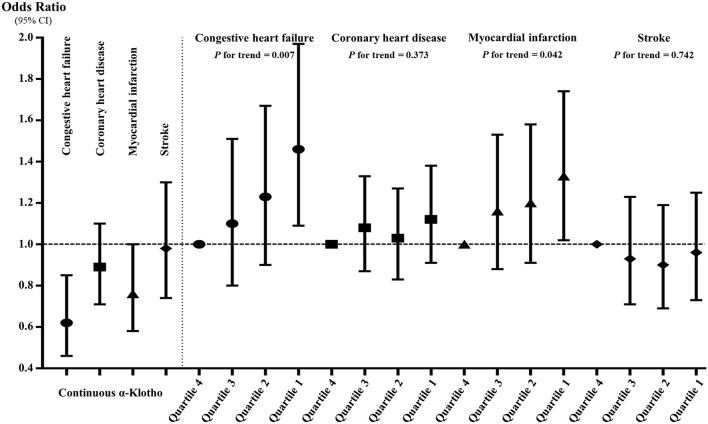
The associations between serum soluble α-Klotho and the four specific cardiovascular diseases in continuous and quartile analyses after full adjustment. The α-Klotho concentrations were the ln-transformed in the continuous analysis.

Figure 3 and Supplementary Table 3 show the associations between α-Klotho and the four specific CVDs. When treating α-Klotho as a continuous variable, the increase in α-Klotho was significantly associated with all study outcomes in Models I and II. In the fully adjusted model, the increase in α-Klotho was only significantly correlated with CHF and MI, indicating that each increment of ln-transformed α-Klotho concentrations was associated with 38 and 24% decreased prevalence of CHF and MI, respectively. When compared to those in the highest quartile, participants in the lowest level of α-Klotho showed generally positive trends for all study outcomes in Models I and II. Similarly, after adjusting for all confounders, participants in the lowest level of Klotho were significantly associated with an increased incidence of CHF (*OR* = 1.46, 95% *CI*: 1.09–1.97, *p* = 0.011) and MI (*OR* = 1.33, 95% *CI*: 1.02–1.74, *p* = 0.037), but it was not the case for CHD (*OR* = 1.12, 95% *CI*: 0.91–1.38, *p* = 0.279) or stroke (*OR* = 0.96, 95% *CI*: 0.73–1.25, *p* = 0.744). Additionally, both in the continuous and quartile analyses, the correlations between α-Klotho and the prevalence of CHF and MI were still significant in Model IV, in which we only adjusted for the significant difference factors in Table 1.

**Figure 4:**
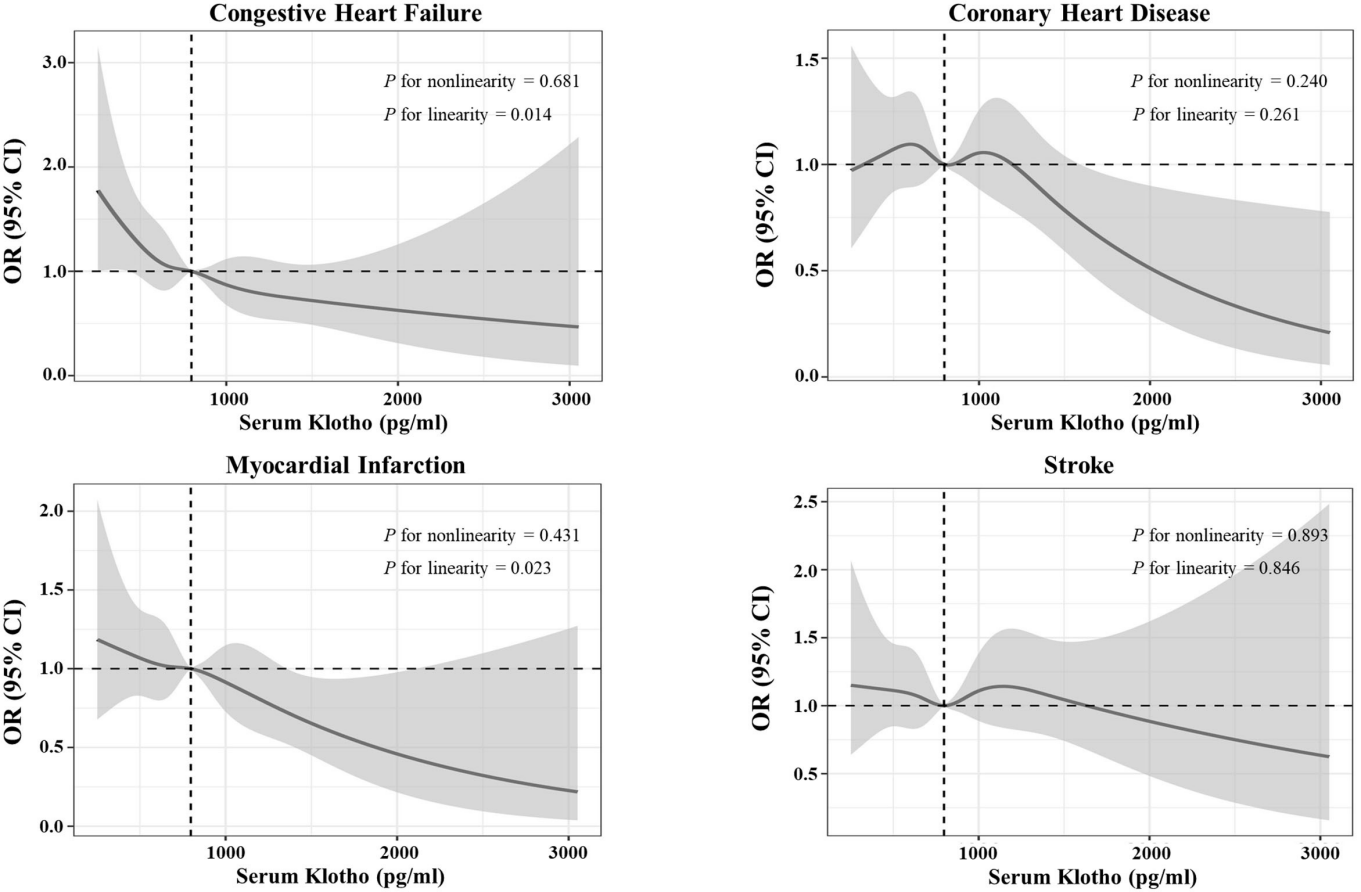
Restricted cubic spine curves for the associations between serum soluble α-Klotho and the four specific cardiovascular diseases. Adjusted odds ratio (*OR*) (solid lines) and 95% confidence intervals (gray shadow) for congestive heart failure (CHF), coronary heart disease, myocardial infarction (MI), and stroke after controlling covariates in Table 1. Spines were examined in the fully adjusted model with the best placed knots at the 5^th^, 35^th^, 65^th^, and 95^th^ percentiles of α-Klotho concentrations.

In Figure 4, we generated restricted cubic spline curves to detect the dose–response effects between α-Klotho and all study outcomes, which indicated that α-Klotho was correlated with CHF and MI in linear-inverse relationships. These associations were consistent with the quartile results in Supplementary Table 3.

### Subgroup Analyses of the Prevalence of Specific CVDs

**Figure 5:**
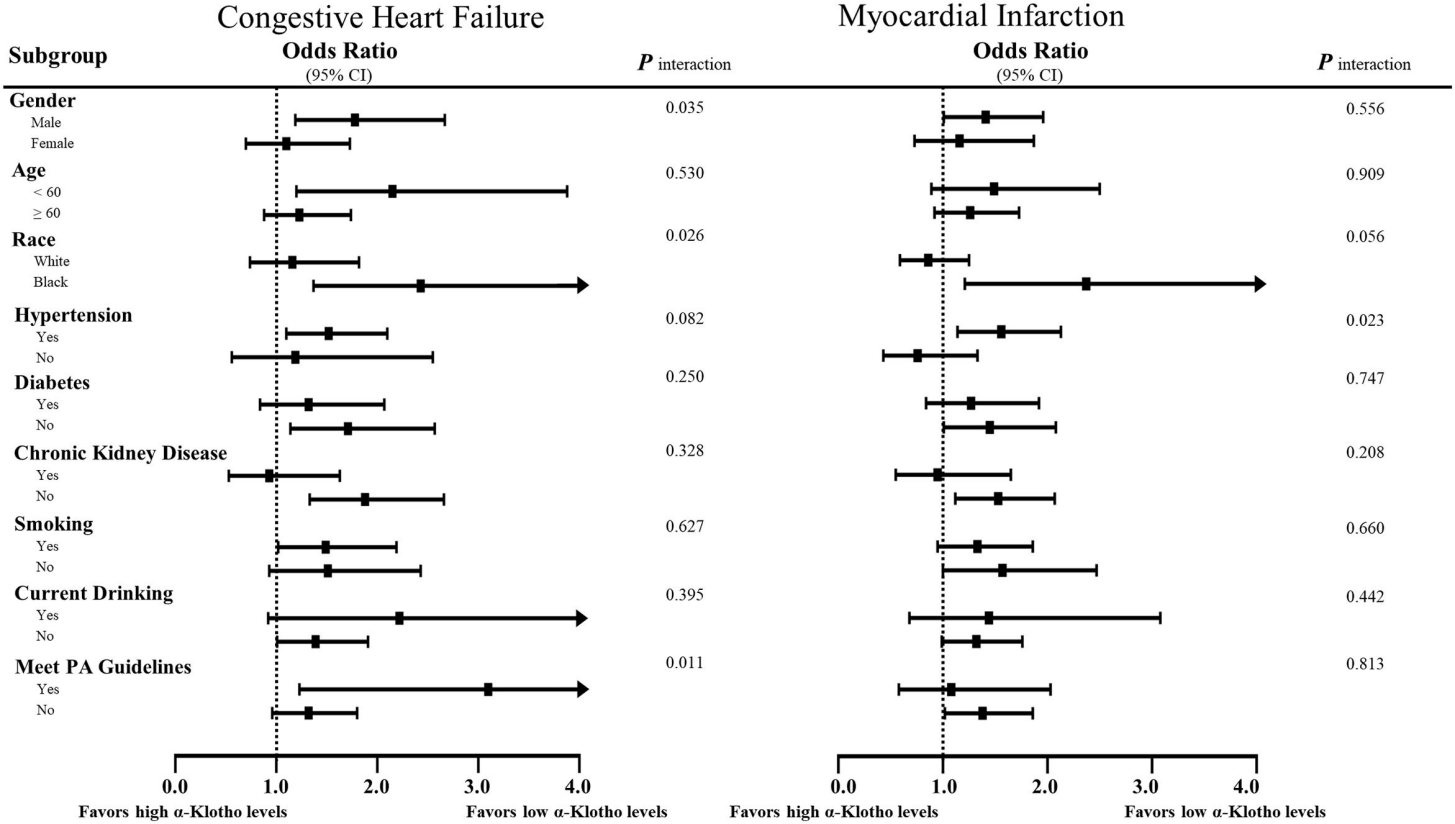
Subgroup analyses of the associations of serum soluble α-Klotho with CHF and MI. Risks of heart failure and MI of the lowest quartile of α-Klotho, as defined by equal to or <646.4 pg/ml, compared to the highest quartile of α-Klotho. The results are expressed as fully adjusted *ORs* after controlling for covariates, such as age, sex, body mass index, race, education, smoking, drinking, diabetes, hypertension, physical activity, white blood cell counts, neutrophil-to-lymphocyte ratio, platelet counts, total cholesterol, triglycerides, low-density lipoprotein cholesterol, high-density lipoprotein cholesterol, serum calcium, serum phosphorus, urine albumin, estimated glomerular filtration rate, HbA1c, energy intake, total fat intake, and medications. In addition, possible interactions between the above factors were also adjusted if necessary.

Stratified multivariate logistic regression analysis was performed for the subgroups of study participants. Compared to the highest quartile, the increased incidence in the lowest quartile was generally significant across some subgroups in CHF and MI but not CHD or stroke (Supplementary Tables 4–7). As shown in Figure 5, the lower α-Klotho concentrations in patients who were male, non-Hispanic Black, hypertensive, diabetes-free, non-drinkers, aged below 60, and had better kidney function indicated that these patients were more susceptible to CHF and MI. Notably, there were some differences in the specific roles of smoking and physical activity level, of which patients who were smokers and had higher physical activity levels were more susceptible to CHF. In addition, a statistical interaction was observed in CHF for sex, race, and physical activity level, whereas interaction with hypertension was observed in MI (Figure 5).

## Discussion

The major findings in the present study were that the lower α-Klotho concentrations are positively associated with an increased prevalence of CHF and MI in linear-inverse relationships, which was not the case for CHD or stroke. After both full adjustments of all potential confounding factors and only adjustment of significant difference factors at baseline, such α-Klotho associations with increased prevalence of CHF and MI remained significant. Moreover, associations were found between each unit increment in the ln-transformed α-Klotho concentrations and a decreased incidence of CHF and MI. Although we observed the interaction of CHF with gender, race, and physical activity level and MI with hypertension, the predictive values of α-Klotho for CHF and MI were generally consistent across participants who were male, non-Hispanic Black, hypertensive, diabetes-free, non-drinkers, and aged below 60 years with better kidney function. To date, explicit experimental evidence supports the cardiovascular and cerebrovascular benefits of α-Klotho (12–15, 22). However, the predictive values and detailed dose–response effects of α-Klotho on specific CVDs have not yet been explored in humans. Collectively, our findings not only further add to the evidence of the cardiovascular impact of α-Klotho but also extend the current knowledge on α-Klotho in humans.

To our knowledge, this is the first report with the largest sample size to better understand the associations between α-Klotho and specific CVDs in humans. Additionally, the present study was supported by the available robust clinical data, particularly multiple traditional CVD risks, including comorbidities, glycolipid metabolism, inflammation markers, nutrition, and lifestyles. Previously, Semba et al. first revealed a negative association between α-Klotho and total CVD among 1,023 members of the general population in Italy (16); intriguingly, they defined the CVD outcome as participants with any CHF, CHD, stroke, or peripheral artery disease, but they did not report a correlation between α-Klotho and a single disease. Compared to the study by Semba et al., we further determined the detailed impact of α-Klotho on specific CVDs with a larger sample and more comprehensive adjustments. Although the study populations in Semba et al. and ours were inconsistent, the knowledge of the potential mechanisms between α-Klotho and CVD has been gradually enriched over the last decade. For example, Keles et al. (23) showed that lower serum α-Klotho levels are associated with increased epicardial fat thickness and carotid artery intima-media thickness and with decreased flow-mediated dilation of the brachial artery. This may be attributed to the negative association of α-Klotho with LDL-C found in the study by Martín-Núñez et al. (24) and the positive correlation with HDL-C observed in the studies by Semba et al. and ours. As mentioned before, α-Klotho upregulates the NO synthesis and improves oxidative stress in human coronary artery endothelial cells (12), and it alleviates cardiac fibrosis without altering cardiomyocyte renewal (14). In addition, α-Klotho inhibits TRPC6 heart channels and regulates myofibroblast activity to protect cardiac hypertrophy and remodeling (25, 26). Furthermore, several studies have indicated that the α-Klotho participates in the modulation of inflammatory cytokines and sterile inflammation in the process of MI and atherosclerotic disease (24, 27). Similarly, the present study observed negative associations between α-Klotho and some inflammatory markers, including the neutrophil-to-lymphocyte ratio, white blood cell count, and platelet count. However, we did not observe correlations of α-Klotho with CHD and stroke. In the present study, we included angina pectoris in the CHD analysis, and there was no significant difference in angina pectoris among the α-Klotho quartile groups, which may be one of the possible reasons why we failed to observe a positive correlation with CHD. A new Mendelian randomization study and meta-analysis have suggested no significant causal association between genetically determined α-Klotho levels and the risks of CHD and stroke (28). CVD is a condition with long-term effects of multiple factors. Due to the lack of raw genetic data in NHANES, we were unable to verify this potential confounder. Interestingly, different study populations may contribute to the paradoxical association between α-Klotho and CHD (28). Thus, it is urgent to investigate other ethnicities to extend our findings. Despite some controversy, our findings suggested that α-Klotho is at least linked to cardiovascular protection. In addition, subgroup analyses indicated that lower α-Klotho concentration in patients who were male, non-Hispanic Black, aged below 60, hypertensive, diabetes-free, non-drinkers, and had better kidney function were more susceptible to CHF and MI. More likely, older patients with comorbidities, such as diabetes and severe chronic kidney disease, are more closely related to metabolic, inflammation, and endothelial dysfunction (5, 29), and the adverse influence of these traditional factors on CVD may be greater than decreased α-Klotho concentrations in some cases. It should also be mentioned that such patients with a higher level of moderate-to-vigorous intensity physical activity were susceptible to CHF, which was not the case for MI. Because CHF is a severe and complex syndrome with fragile cardiac function, current guidelines recommend that patients with CHF engage in a maximum of 180 min per week of moderate-intensity physical activity at 80% peak VO_2_ (19, 20). In the present study, we classified the higher physical activity level based on at least 150 min per week of moderate and/or 75 min of vigorous physical activity for the general population, surpassing the recommendation for patients with CHF. Thus, the result that lower the α-Klotho concentrations in patients engaged in higher levels of physical activity indicated a greater association with increased CHF was in line with the guidelines. However, although we observed that the Klotho concentrations did not differ according to physical activity level, a higher level of physical activity may offset the harm from lower α-Klotho concentrations, suggesting that higher levels of physical activity should also be recommended for patients to prevent MI. Importantly, the detailed mechanism underlying the interaction between the above conventional risk factors and α-Klotho on human cardiovascular impacts merits further investigation.

The present study had several limitations. First, the outcomes of CVD were self-reported diagnoses from the questionnaire, leading to recall bias. Second, given the nature of an observational cross-sectional study, we were unable to draw causal conclusions regarding the associations between α-Klotho and specific CVDs in humans. However, the α-Klotho was established as an independent predictive biomarker for CHF and MI. Third, we did not obtain detailed causes of study outcomes, such as hemorrhagic or ischemic stroke, from NHANES, suggesting that hemorrhagic stroke may be included in the study. However, there is no existing evidence supporting the association of α-Klotho with hemorrhagic stroke, which may explain why there was no significant association between α-Klotho and stroke in the present study. Finally, the α-Klotho was measured at baseline, and the fluctuation of individual α-Klotho levels was not considered in our study, which prevented further investigation of the time-course associations between changes in α-Klotho concentrations and the incidence of new CVD events. Thus, the results should be interpreted with caution in clinical practice.

## Conclusion

The present cross-sectional study with a large sample size provided supportive evidence of positive associations of α-Klotho with CHF and MI. Additionally, linear-inverse associations between α-Klotho and the prevalence of CHF and MI were observed. Further well-designed prospective studies are necessary to determine the specific effects of α-Klotho on different subtypes of CVD, especially CHD and stroke, in humans.

## Data Availability Statement

The original contributions presented in the study are included in the article/Supplementary Materials, further inquiries can be directed to the corresponding author.

## Ethics Statement

The studies involving human participants were reviewed and approved by National Center for Health Statistics. The patients/participants provided their written informed consent to participate in this study.

## Author Contributions

J-PX, R-XZ, M-HH, S-SL, L-HG, and M-ZZ conceived and designed the study. J-PX, R-XZ, S-SL, and M-HH collected and analyzed the data. J-PX drafted the paper. M-ZZ revised the manuscript. All authors have reviewed the final manuscript.

## Funding

This research was funded by the Team for the Prevention and Treatment of Acute Myocardial Infarction with Chinese Medicine (2019KCXTD009 to M-ZZ), the National Natural Scientific Foundation (Nos. 81703848, 81703877, and 82004135), and the Research Fund for Zhaoyang Talents of Guangdong Provincial Hospital of Chinese Medicine (No. ZY2022KY03).

## Conflict of Interest

The authors declare that the research was conducted in the absence of any commercial or financial relationships that could be construed as a potential conflict of interest.

## Publisher's Note

All claims expressed in this article are solely those of the authors and do not necessarily represent those of their affiliated organizations, or those of the publisher, the editors and the reviewers. Any product that may be evaluated in this article, or claim that may be made by its manufacturer, is not guaranteed or endorsed by the publisher.
