# Supplementary material for: Associations Between Serum Soluble α-Klotho and the Prevalence of Specific Cardiovascular Disease
Source: Front Cardiovasc Med. 2022 Jun 20;9:899307. doi: 10.3389/fcvm.2022.899307 (PMC9251131; doi:10.3389/fcvm.2022.899307)
Supplement: Supplementary file 2 [file Data_Sheet_2.docx]

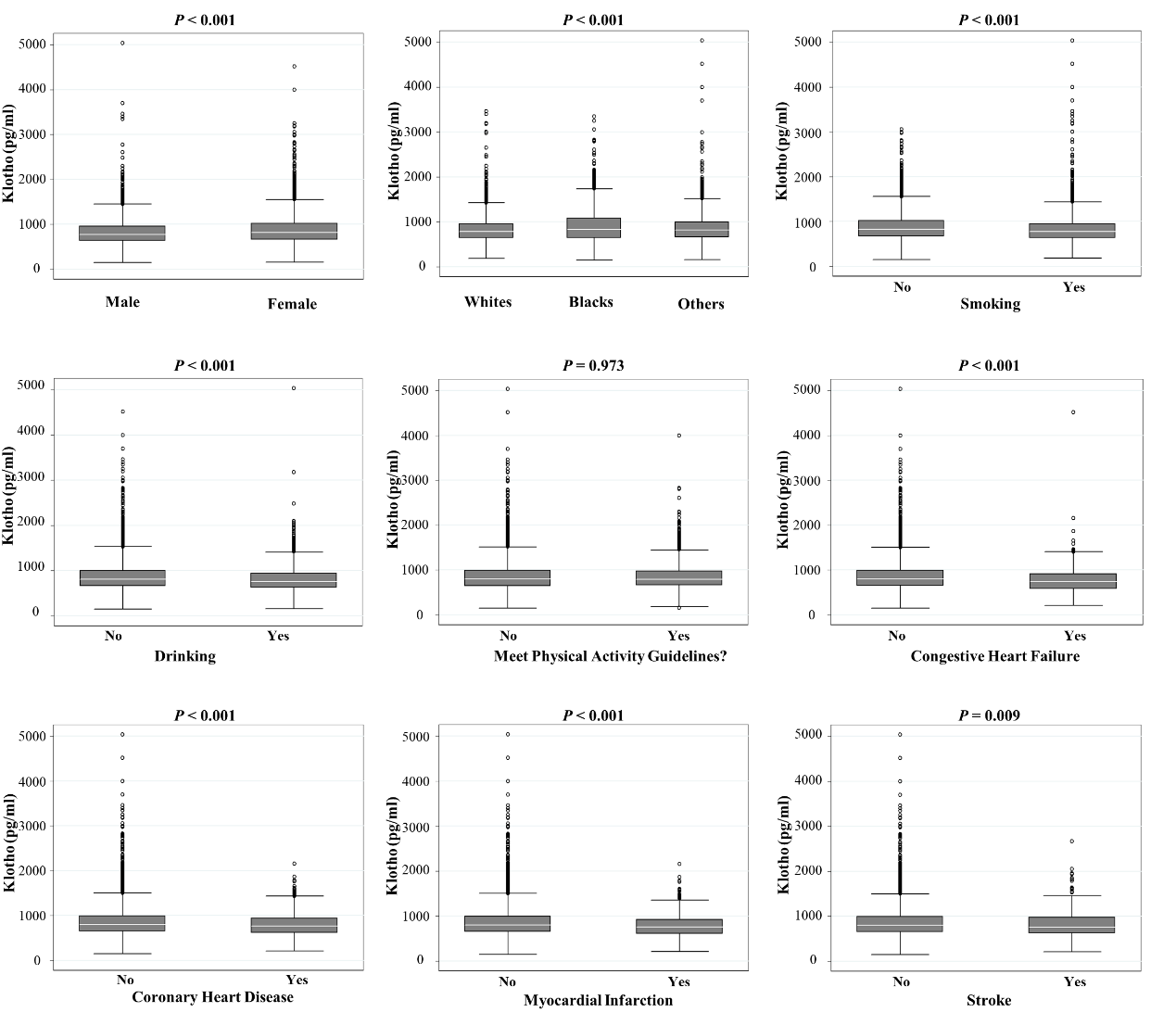


Figure S1. α-Klotho concentrations by categorical participant characteristics and prespecified outcomes. Box plots for α-Klotho for sex, race/ethnicity, smoking status, current smoking status, physical activity level, congestive heart failure, coronary heart disease, myocardial infarction and stroke. The middle white line of the box represents the median value with the edges of the box representing the 25th and 75th percentiles. The whiskers extend to 1.5× (interquartile range), and the dots represent values falling outside that range. *P* *value* determined by Kruskal–Wallis tests that treat continuous serum α-Klotho concentrations as the dependent variable and the participant characteristic as the independent variable.

| Table S1. *P* *values* of the Spearman correlation analysis between baseline continuous variables and serum α-Klotho concentrations | | | | | | | | | | | | | | |  |
| --- | --- | --- | --- | --- | --- | --- | --- | --- | --- | --- | --- | --- | --- | --- | --- |
|  | α-Klotho | Age | BMI | eGFR | WBC | NLR | Platelet | HbA1c | HDL-C | TC | Triglyceride | LDL-C | Energy intake | Total fat intake |  |
| α-Klotho | ＜0.0001 | ＜0.0001 | 0.1041 | ＜0.0001 | ＜0.0001 | ＜0.0001 | ＜0.0001 | 0.1319 | 0.023 | 0.2257 | ＜0.0001 | 0.0317 | 0.5256 | 0.4672 |  |
| Age | ＜0.0001 | ＜0.0001 | ＜0.0001 | ＜0.0001 | ＜0.0001 | ＜0.0001 | ＜0.0001 | ＜0.0001 | ＜0.0001 | ＜0.0001 | 0.6093 | ＜0.0001 | ＜0.0001 | ＜0.0001 |  |
| BMI | 0.1041 | ＜0.0001 | ＜0.0001 | 0.102 | ＜0.0001 | 0.0177 | ＜0.0001 | ＜0.0001 | ＜0.0001 | ＜0.0001 | ＜0.0001 | 0.126 | 0.0733 | ＜0.0001 |  |
| eGFR | ＜0.0001 | ＜0.0001 | 0.102 | ＜0.0001 | 0.2661 | ＜0.0001 | ＜0.0001 | ＜0.0001 | 0.0043 | ＜0.0001 | 0.0001 | ＜0.0001 | ＜0.0001 | ＜0.0001 |  |
| WBC | ＜0.0001 | ＜0.0001 | ＜0.0001 | 0.2661 | ＜0.0001 | ＜0.0001 | ＜0.0001 | ＜0.0001 | ＜0.0001 | 0.0004 | ＜0.0001 | 0.0007 | 0.3776 | 0.3257 |  |
| NLR | ＜0.0001 | ＜0.0001 | 0.0177 | ＜0.0001 | ＜0.0001 | ＜0.0001 | 0.0775 | 0.0908 | ＜0.0001 | ＜0.0001 | 0.8458 | ＜0.0001 | 0.0011 | 0.0001 |  |
| Platelet | ＜0.0001 | ＜0.0001 | ＜0.0001 | ＜0.0001 | ＜0.0001 | 0.0775 | ＜0.0001 | 0.0072 | 0.0011 | ＜0.0001 | ＜0.0001 | ＜0.0001 | ＜0.0001 | ＜0.0001 |  |
| HbA1c | 0.1319 | ＜0.0001 | ＜0.0001 | ＜0.0001 | ＜0.0001 | 0.0908 | 0.0072 | ＜0.0001 | ＜0.0001 | ＜0.0001 | ＜0.0001 | ＜0.0001 | ＜0.0001 | 0.002 |  |
| HDL-C | 0.023 | ＜0.0001 | ＜0.0001 | 0.0043 | ＜0.0001 | ＜0.0001 | 0.0011 | ＜0.0001 | ＜0.0001 | ＜0.0001 | ＜0.0001 | ＜0.0001 | ＜0.0001 | ＜0.0001 |  |
| Total cholesterol | 0.2257 | ＜0.0001 | ＜0.0001 | ＜0.0001 | 0.0004 | ＜0.0001 | ＜0.0001 | ＜0.0001 | ＜0.0001 | ＜0.0001 | ＜0.0001 | ＜0.0001 | 0.0757 | 0.0327 |  |
| Triglyceride | ＜0.0001 | 0.6093 | ＜0.0001 | 0.0001 | ＜0.0001 | 0.8458 | ＜0.0001 | ＜0.0001 | ＜0.0001 | ＜0.0001 | ＜0.0001 | ＜0.0001 | 0.9149 | 0.059 |  |
| LDL-C | 0.0317 | ＜0.0001 | 0.126 | ＜0.0001 | 0.0007 | ＜0.0001 | ＜0.0001 | ＜0.0001 | ＜0.0001 | ＜0.0001 | ＜0.0001 | ＜0.0001 | 0.7679 | 0.9751 |  |
| Energy intake | 0.5256 | ＜0.0001 | 0.0733 | ＜0.0001 | 0.3776 | 0.0011 | ＜0.0001 | ＜0.0001 | ＜0.0001 | 0.0757 | 0.9149 | 0.7679 | ＜0.0001 | ＜0.0001 |  |
| Total fat intake | 0.4672 | ＜0.0001 | ＜0.0001 | ＜0.0001 | 0.3257 | 0.0001 | ＜0.0001 | 0.002 | ＜0.0001 | 0.0327 | 0.059 | 0.9751 | ＜0.0001 | ＜0.0001 |  |
| BMI, body mass index; eGFR, estimated glomerular filtration rate; WBC, white blood cell; NLR, neutrophil-to-lymphocyte ratio; HbA1c, glycated haemoglobin; HDL-C, high-density lipoprotein cholesterol; TC, total cholesterol; LDL-C, low-density lipoprotein cholesterol. | | | | | | | | | | | | | | |  |
|  |  |  |  |  |  |  |  |  |  |  |  |  |  |  |  |
|  |  |  |  |  |  |  |  |  |  |  |  |  |  |  |  |

| Table S2. Serum α-Klotho concentrations according to baseline categorical variables and prespecified outcomes. | | | |  |
| --- | --- | --- | --- | --- |
|  |  |  |  |  |
|  | Mean (SD) | Median (IQR) | *P* *value* |  |
| Sex |  |  | < 0.001 |  |
| Male | 826.0 (302.0) | 773.6 (633.0-960.7) |  |  |
| Female | 871.7 (330.4) | 816.3 (659.5-1015.0) |  |  |
| Race |  |  | < 0.001 |  |
| Non-Hispanic White | 823.6 (280.4) | 779.5 (643.8-955.1) |  |  |
| Non-Hispanic Black | 899.8 (378.8) | 822.8 (643.5-1082.8) |  |  |
| Other | 861.2 (327.0) | 812.8 (655.0-996.9) |  |  |
| Smoking Status |  |  | < 0.001 |  |
| Never | 876.0 (315.3) | 825.6 (664.4-1021.2) |  |  |
| Ever & Current | 826.2 (319.6) | 769.8 (629.5-952.0) |  |  |
| Current Drinking |  |  | < 0.001 |  |
| No | 862.5 (322.9) | 808.9 (654.9-1004.7) |  |  |
| Yes | 807.9 (297.9) | 758.1 (621.1-936.4) |  |  |
| Meet Physical Activity Guidelines? |  |  | 0.973 |  |
| No | 851.6 (324.2) | 799.2 (642.5-992.0) |  |  |
| Yes | 847.9 (301.2) | 789.8 (656.0-976.4) |  |  |
| Congestive heart failure |  |  | < 0.001 |  |
| No | 854.6 (318.2) | 800.3 (650.7-991.4) |  |  |
| Yes | 780.3 (315.4) | 741.3 (579.8-909.9) |  |  |
| Coronary heart disease |  |  | < 0.001 |  |
| No | 856.9 (324.5) | 801.2 (652.4-993.6) |  |  |
| Yes | 805.3 (266.0) | 766.2 (613.3-940.7) |  |  |
| Myocardial infarction |  |  | < 0.001 |  |
| No | 855.4 (321.8) | 800.7 (650.8-992.5) |  |  |
| Yes | 786.9 (261.7) | 751.9 (606.5-918.0) |  |  |
| Stroke |  |  | 0.009 |  |
| No | 852.8 (319.5) | 798.2 (648.9-988.8) |  |  |
| Yes | 815.8 (298.8) | 757.5 (624.1-973.0) |  |  |
| SD, standard deviation; IQR, interquartile range. | | | |  |

| Table S3. Association between serum α-Klotho concentrations and the specific cardiovascular disease | | | | |  |
| --- | --- | --- | --- | --- | --- |
|  | Congestive heart failure | Coronary heart disease | Myocardial infarction | Stroke |  |
| Model I^a^ |  |  |  |  |  |
| Continuous α-Klotho^e^ | **0.44 (0.33, 0.58) < 0.001** | **0.62 (0.52, 0.76) < 0.001** | **0.51 (0.40, 0.66) < 0.001** | **0.66 (0.51, 0.86) 0.002** |  |
| Categories |  |  |  |  |  |
| Quartile 4 | Reference | Reference | Reference | Reference |  |
| Quartile 3 | 1.08 (0.81, 1.45) 0.599 | 1.16 (0.96, 1.41) 0.130 | 1.25 (0.97, 1.62) 0.090 | 0.97 (0.75, 1.27) 0.839 |  |
| Quartile 2 | 1.26 (0.95, 1.68) 0.111 | 1.21 (1.00, 1.46) 0.052 | **1.41 (1.09, 1.81) 0.008** | 1.01 (0.78, 1.31) 0.943 |  |
| Quartile 1 | **1.92 (1.48, 2.51) < 0.001** | **1.53 (1.27, 1.84) < 0.001** | **1.86 (1.46, 2.37) < 0.001** | **1.34 (1.05, 1.72) 0.020** |  |
| *P* for trend | < 0.001 | < 0.001 | < 0.001 | 0.018 |  |
| Model II^b^ |  |  |  |  |  |
| Continuous α-Klotho^e^ | **0.52 (0.39, 0.68) < 0.001** | **0.78 (0.64, 0.95) 0.012** | **0.64 (0.50, 0.83) < 0.001** | **0.74 (0.57, 0.97) 0.031** |  |
| Categories |  |  |  |  |  |
| Quartile 4 | Reference | Reference | Reference | Reference |  |
| Quartile 3 | 1.07 (0.79, 1.44) 0.662 | 1.08 (0.89, 1.32) 0.421 | 1.16 (0.89, 1.51) 0.263 | 0.98 (0.75, 1.28) 0.857 |  |
| Quartile 2 | 1.19 (0.89, 1.59) 0.245 | 1.05 (0.87, 1.28) 0.604 | 1.21 (0.94, 1.57) 0.141 | 0.98 (0.75, 1.28) 0.894 |  |
| Quartile 1 | **1.70 (1.30, 2.22) < 0.001** | **1.27 (1.05, 1.53) 0.014** | **1.53 (1.20, 1.96) < 0.001** | 1.22 (0.95, 1.57) 0.124 |  |
| *P* for trend | < 0.001 | 0.020 | < 0.001 | 0.119 |  |
| Model III^c^ |  |  |  |  |  |
| Continuous α-Klotho^e^ | **0.62 (0.46, 0.84) 0.002** | 0.89 (0.71, 1.10) 0.285 | **0.76 (0.57, 1.00) 0.048** | 0.98 (0.74, 1.30) 0.891 |  |
| Categories |  |  |  |  |  |
| Quartile 4 | Reference | Reference | Reference | Reference |  |
| Quartile 3 | 1.11 (0.81, 1.52) 0.535 | 1.08 (0.87, 1.34) 0.471 | 1.16 (0.88, 1.53) 0.289 | 0.93 (0.71, 1.23) 0.628 |  |
| Quartile 2 | 1.23 (0.90, 1.67) 0.192 | 1.03 (0.83, 1.27) 0.791 | 1.20 (0.91, 1.58) 0.199 | 0.90 (0.69, 1.19) 0.479 |  |
| Quartile 1 | **1.46 (1.09, 1.97) 0.011** | 1.12 (0.91, 1.38) 0.279 | **1.33 (1.02, 1.74) 0.037** | 0.96 (0.73, 1.25) 0.744 |  |
| *P* for trend | 0.007 | 0.373 | 0.041 | 0.742 |  |
| Model IV^d^ |  |  |  |  |  |
| Continuous α-Klotho^e^ | **0.63 (0.47, 0.85) 0.003** | 0.89 (0.71, 1.10) 0.284 | **0.76 (0.58, 1.00) 0.050** | 0.98 (0.74, 1.30) 0.889 |  |
| Categories |  |  |  |  |  |
| Quartile 4 | Reference | Reference | Reference | Reference |  |
| Quartile 3 | 1.13 (0.83, 1.55) 0.444 | 1.09 (0.88, 1.35) 0.433 | 1.17 (0.88, 1.54) 0.276 | 0.93 (0.71, 1.23) 0.616 |  |
| Quartile 2 | 1.23 (0.91, 1.68) 0.180 | 1.03 (0.83, 1.27) 0.795 | 1.18 (0.90, 1.55) 0.230 | 0.90 (0.68, 1.18) 0.443 |  |
| Quartile 1 | **1.45 (1.08, 1.95) 0.013** | 1.12 (0.91, 1.38) 0.280 | **1.32 (1.01, 1.73) 0.040** | 0.96 (0.73, 1.25) 0.737 |  |
| *P* for trend | 0.010 | 0.387 | 0.048 | 0.730 |  |
| ^a^Crude model;  ^b^Adjusted for age, gender and race;  ^c^Adjusted models were adjusted for age, gender, body mass index, race, education, smoking, drinking, diabetes, hypertension, physical activity, white blood cell counts, neutrophil-to-lymphocyte ratio, platelet counts, total cholesterol, triglyceride, low-density lipoprotein cholesterol, high-density lipoprotein cholesterol, serum calcium, serum phosphorus, urine albumin, estimated glomerular filtration rate, HbA1c, energy intake, total fat intake and medications;  ^d^Only adjusted for significant difference factors in Table 1;  ^e^Continuous variables were ln-transformed. | | | | |  |
|  |  |  |  |  |  |
|  |  |  |  |  |  |
|  |  |  |  |  |  |
|  |  |  |  |  |  |
|  |  |  |  |  |  |

| Table S4. Odds ratios (and 95% confidence intervals) for the association of serum α-Klotho concentrations with heart failure, stratified by participant characteristics | | | | | |  |
| --- | --- | --- | --- | --- | --- | --- |
|  |  |  |  |  |  |  |
|  | Fully adjusted OR (95% CI) | *P* *value* | Fully adjusted OR (95% CI) | *P* *value* | *P* interaction |  |
| Categories | Male (n = 3,967) |  | Female (n = 4,648) |  | 0.036 |  |
| Quartile 4 | Reference | Reference | Reference | Reference |  |  |
| Quartile 3 | 1.11 (0.71, 1.73) | 0.661 | 1.13 (0.72, 1.76) | 0.595 |  |  |
| Quartile 2 | 1.42 (0.93, 2.17) | 0.104 | 1.01 (0.64, 1.61) | 0.958 |  |  |
| Quartile 1 | **1.78 (1.19, 2.67)** | 0.005 | 1.10 (0.70, 1.73) | 0.668 |  |  |
| *P* for trend |  | 0.002 |  | 0.789 |  |  |
|  | <60 years old (n = 3,871) |  | ≥60 years old (n = 4,744) |  | 0.530 |  |
| Quartile 4 | Reference | Reference | Reference | Reference |  |  |
| Quartile 3 | 1.47 (0.79, 2.72) | 0.225 | 1.00 (0.69, 1.44) | 0.989 |  |  |
| Quartile 2 | 1.42 (0.76, 2.65) | 0.270 | 1.13 (0.79, 1.62) | 0.499 |  |  |
| Quartile 1 | **2.15 (1.20, 3.88)** | 0.011 | 1.23 (0.88, 1.74) | 0.230 |  |  |
| *P* for trend |  | 0.015 |  | 0.158 |  |  |
|  | Non-Hispanic White (n = 4,207) |  | Non-Hispanic Black (n = 1,753) |  | 0.026 |  |
| Quartile 4 | Reference | Reference | Reference | Reference |  |  |
| Quartile 3 | 1.09 (0.69, 1.73) | 0.715 | 1.26 (0.65, 2.45) | 0.490 |  |  |
| Quartile 2 | 1.14 (0.72, 1.81) | 0.587 | **2.06 (1.12, 3.79)** | 0.020 |  |  |
| Quartile 1 | 1.16 (0.74, 1.82) | 0.521 | **2.43 (1.37, 4.32)** | 0.003 |  |  |
| *P* for trend |  | 0.521 |  | < 0.001 |  |  |
|  | Hypertension (n = 5,204) |  | Non-Hypertension (n = 3,411) |  | 0.082 |  |
| Quartile 4 | Reference | Reference | Reference | Reference |  |  |
| Quartile 3 | 1.04 (0.73, 1.47) | 0.840 | 1.34 (0.63, 2.85 | 0.445 |  |  |
| Quartile 2 | 1.32 (0.94, 1.84) | 0.109 | 0.86 (0.38, 1.97) | 0.727 |  |  |
| Quartile 1 | **1.52 (1.10, 2.10)** | 0.011 | 1.19 (0.56, 2.55) | 0.653 |  |  |
| *P* for trend |  | 0.004 |  | 0.922 |  |  |
|  | Diabetes (n = 2,090) |  | Non-Diabetes (n = 6,525) |  | 0.250 |  |
| Quartile 4 | Reference | Reference | Reference | Reference |  |  |
| Quartile 3 | 0.72 (0.43, 1.22) | 0.221 | 1.49 (0.98, 2.26) | 0.059 |  |  |
| Quartile 2 | 1.17 (0.72, 1.88) | 0.531 | 1.40 (0.92, 2.14) | 0.116 |  |  |
| Quartile 1 | 1.32 (0.84, 2.07) | 0.228 | **1.71 (1.14, 2.57)** | 0.010 |  |  |
| *P* for trend |  | 0.085 |  | 0.022 |  |  |
|  | Chronic kidney disease (n = 1,115) |  | Nonchronic kidney disease (n = 7,500) |  | 0.328 |  |
| Quartile 4 | Reference | Reference | Reference | Reference |  |  |
| Quartile 3 | 0.72 (0.37, 1.42) | 0.348 | 1.27 (0.89, 1.83) | 0.187 |  |  |
| Quartile 2 | 1.32 (0.73, 2.38) | 0.364 | 1.21 (0.83, 1.75) | 0.323 |  |  |
| Quartile 1 | 0.93 (0.53, 1.63) | 0.797 | **1.88 (1.33, 2.66)** | 0.000 |  |  |
| *P* for trend |  | 0.819 |  | < 0.001 |  |  |
|  | Ever & Current Smoking (n = 4,232) |  | Never Smoking (n = 4,383) |  | 0.627 |  |
| Quartile 4 | Reference | Reference | Reference | Reference |  |  |
| Quartile 3 | 1.03 (0.68, 1.56) | 0.878 | 1.26 (0.77, 2.08) | 0.359 |  |  |
| Quartile 2 | 1.21 (0.81, 1.80) | 0.351 | 1.35 (0.82, 2.24) | 0.235 |  |  |
| Quartile 1 | **1.49 (1.02, 2.19)** | 0.041 | 1.51 (0.93, 2.43) | 0.092 |  |  |
| *P* for trend |  | 0.020 |  | 0.094 |  |  |
|  | Current Drinking (n = 1,866) |  | Noncurrent Drinking (n = 6,749) |  | 0.395 |  |
| Quartile 4 | Reference | Reference | Reference | Reference |  |  |
| Quartile 3 | 1.52 (0.57, 4.04) | 0.401 | 1.08 (0.77, 1.51) | 0.661 |  |  |
| Quartile 2 | 1.76 (0.70, 4.44) | 0.227 | 1.19 (0.86, 1.67) | 0.294 |  |  |
| Quartile 1 | 2.22 (0.92, 5.35) | 0.075 | **1.39 (1.01, 1.91)** | 0.044 |  |  |
| *P* for trend |  | 0.064 |  | 0.033 |  |  |
|  | Meet PA Guidelines (n = 2,211) |  | Did Not Meet PA Guidelines (n = 6,404) |  | 0.011 |  |
| Quartile 4 | Reference | Reference | Reference | Reference |  |  |
| Quartile 3 | 0.94 (0.29, 3.02) | 0.919 | 1.12 (0.80, 1.55) | 0.512 |  |  |
| Quartile 2 | 2.46 (0.97, 6.26) | 0.058 | 1.12 (0.80, 1.56) | 0.521 |  |  |
| Quartile 1 | **3.10 (1.23, 7.83)** | 0.016 | 1.32 (0.96, 1.80) | 0.087 |  |  |
| *P* for trend |  | 0.003 |  | 0.095 |  |  |
| PA, physical activity. | | | | |  |  |

| Table S5. Odds ratios (and 95% confidence intervals) for the association of serum α-Klotho concentrations with coronary heart disease, stratified by participant characteristics | | | | | |  |
| --- | --- | --- | --- | --- | --- | --- |
|  |  |  |  |  |  |  |
|  | Fully adjusted OR (95% CI) | *P* *value* | Fully adjusted OR (95% CI) | *P* *value* | *P* interaction |  |
| Categories | Male (n = 3,967) |  | Female (n = 4,648) |  | 0.177 |  |
| Quartile 4 | Reference | Reference | Reference | Reference |  |  |
| Quartile 3 | 1.32 (0.99, 1.76) | 0.061 | 0.84 (0.61, 1.16) | 0.292 |  |  |
| Quartile 2 | 1.15 (0.87, 1.54) | 0.328 | 0.90 (0.65, 1.24) | 0.516 |  |  |
| Quartile 1 | 1.31 (0.99, 1.74) | 0.059 | 0.93 (0.67, 1.28) | 0.658 |  |  |
| *P* for trend |  | 0.156 |  | 0.779 |  |  |
|  | <60 years old (n = 3,871) |  | ≥60 years old (n = 4,744) |  | 0.704 |  |
| Quartile 4 | Reference | Reference | Reference | Reference |  |  |
| Quartile 3 | 1.07 (0.72, 1.58) | 0.738 | 1.08 (0.84, 1.40) | 0.533 |  |  |
| Quartile 2 | 1.02 (0.68, 1.52) | 0.930 | 1.01 (0.79, 1.31) | 0.921 |  |  |
| Quartile 1 | 1.26 (0.85, 1.87) | 0.252 | 1.07 (0.83, 1.37) | 0.612 |  |  |
| *P* for trend |  | 0.312 |  | 0.767 |  |  |
|  | Non-Hispanic White (n = 4,207) |  | Non-Hispanic Black (n = 1,753) |  | 0.166 |  |
| Quartile 4 | Reference | Reference | Reference | Reference |  |  |
| Quartile 3 | 1.03 (0.76, 1.40) | 0.844 | 1.27 (0.79, 2.04) | 0.333 |  |  |
| Quartile 2 | 0.83 (0.61, 1.13) | 0.237 | 1.56 (0.99, 2.47) | 0.057 |  |  |
| Quartile 1 | 0.83 (0.61, 1.13) | 0.240 | 1.17 (0.74, 1.85) | 0.503 |  |  |
| *P* for trend |  | 0.109 |  | 0.371 |  |  |
|  | Hypertension (n = 5,204) |  | Non-Hypertension (n = 3,411) |  | 0.549 |  |
| Quartile 4 | Reference | Reference | Reference | Reference |  |  |
| Quartile 3 | 1.13 (0.89, 1.45) | 0.312 | 0.88 (0.56, 1.37) | 0.573 |  |  |
| Quartile 2 | 1.17 (0.92, 1.49) | 0.204 | 0.67 (0.42, 1.08) | 0.100 |  |  |
| Quartile 1 | 1.20 (0.94, 1.52) | 0.138 | 0.84 (0.53, 1.34) | 0.464 |  |  |
| *P* for trend |  | 0.151 |  | 0.329 |  |  |
|  | Diabetes (n = 2,090) |  | Non-Diabetes (n = 6,525) |  | 0.714 |  |
| Quartile 4 | Reference | Reference | Reference | Reference |  |  |
| Quartile 3 | 1.17 (0.82, 1.67) | 0.396 | 1.07 (0.82, 1.40) | 0.619 |  |  |
| Quartile 2 | 1.16 (0.81, 1.66) | 0.414 | 1.00 (0.77, 1.32) | 0.979 |  |  |
| Quartile 1 | 1.19 (0.84, 1.68) | 0.318 | 1.09 (0.84, 1.43) | 0.513 |  |  |
| *P* for trend |  | 0.361 |  | 0.639 |  |  |
|  | Chronic kidney disease (n = 1,115) |  | Nonchronic kidney disease (n = 7,500) |  | 0.126 |  |
| Quartile 4 | Reference | Reference | Reference | Reference |  |  |
| Quartile 3 | 0.93 (0.55, 1.59) | 0.797 | 1.14 (0.90, 1.44) | 0.280 |  |  |
| Quartile 2 | 0.68 (0.41, 1.13) | 0.133 | 1.16 (0.91, 1.47) | 0.226 |  |  |
| Quartile 1 | 0.75 (0.47, 1.20) | 0.237 | **1.28 (1.01, 1.62)** | 0.040 |  |  |
| *P* for trend |  | 0.164 |  | 0.047 |  |  |
|  | Ever & Current Smoking (n = 4,232) |  | Never Smoking (n = 4,383) |  | 0.466 |  |
| Quartile 4 | Reference | Reference | Reference | Reference |  |  |
| Quartile 3 | 1.17 (0.89, 1.54) | 0.272 | 0.92 (0.65, 1.30) | 0.651 |  |  |
| Quartile 2 | 1.00 (0.76, 1.32) | 0.994 | 1.14 (0.81, 1.60) | 0.470 |  |  |
| Quartile 1 | 1.07 (0.81, 1.40) | 0.632 | 1.32 (0.95, 1.84) | 0.101 |  |  |
| *P* for trend |  | 0.975 |  | 0.054 |  |  |
|  | Current Drinking (n = 1,866) |  | Noncurrent Drinking (n = 6,749) |  | 0.391 |  |
| Quartile 4 | Reference | Reference | Reference | Reference |  |  |
| Quartile 3 | 1.58 (0.90, 2.78) | 0.110 | 1.00 (0.79, 1.26) | 0.986 |  |  |
| Quartile 2 | 1.24 (0.72, 2.15) | 0.436 | 1.00 (0.79, 1.27) | 0.987 |  |  |
| Quartile 1 | 1.08 (0.62, 1.88) | 0.781 | 1.14 (0.90, 1.43) | 0.275 |  |  |
| *P* for trend |  | 0.779 |  | 0.276 |  |  |
|  | Meet PA Guidelines (n = 2,211) |  | Did Not Meet PA Guidelines (n = 6,404) |  | 0.998 |  |
| Quartile 4 | Reference | Reference | Reference | Reference |  |  |
| Quartile 3 | 1.30 (0.80, 2.12) | 0.293 | 1.03 (0.82, 1.31) | 0.777 |  |  |
| Quartile 2 | 1.01 (0.63, 1.64) | 0.957 | 1.03 (0.81, 1.31) | 0.827 |  |  |
| Quartile 1 | 1.17 (0.72, 1.90) | 0.525 | 1.10 (0.87, 1.38) | 0.443 |  |  |
| *P* for trend |  | 0.810 |  | 0.466 |  |  |
| PA, physical activity. | | | | |  |  |

| Supplemental Table 6. Odds ratios (and 95% confidence intervals) for the association of serum α-Klotho concentrations with myocardial infarction, stratified by participant characteristics | | | | | |  |
| --- | --- | --- | --- | --- | --- | --- |
|  |  |  |  |  |  |  |
|  | Fully adjusted OR (95% CI) | *P* *value* | Fully adjusted OR (95% CI) | *P* *value* | *P* interaction |  |
| Categories | Male (n = 3,967) |  | Female (n = 4,648) |  | 0.556 |  |
| Quartile 4 | Reference | Reference | Reference | Reference |  |  |
| Quartile 3 | 1.04 (0.73, 1.48) | 0.814 | 1.36 (0.86, 2.15) | 0.130 |  |  |
| Quartile 2 | 1.14 (0.81, 1.60) | 0.465 | 1.30 (0.81, 2.07) | 0.940 |  |  |
| Quartile 1 | **1.41 (1.01, 1.96)** | 0.043 | 1.16 (0.73, 1.87) | 0.360 |  |  |
| *P* for trend |  | 0.028 |  | 0.670 |  |  |
|  | <60 years old (n = 3,871) |  | ≥60 years old (n = 4,744) |  | 0.909 |  |
| Quartile 4 | Reference | Reference | Reference | Reference |  |  |
| Quartile 3 | 1.23 (0.73, 2.07) | 0.439 | 1.12 (0.81, 1.57) | 0.488 |  |  |
| Quartile 2 | 1.33 (0.79, 2.23) | 0.280 | 1.14 (0.82, 1.58) | 0.432 |  |  |
| Quartile 1 | 1.49 (0.89, 2.50) | 0.132 | 1.26 (0.92, 1.73) | 0.154 |  |  |
| *P* for trend |  | 0.130 |  | 0.165 |  |  |
|  | Non-Hispanic White (n = 4,207) |  | Non-Hispanic Black (n = 1,753) |  | 0.056 |  |
| Quartile 4 | Reference | Reference | Reference | Reference |  |  |
| Quartile 3 | 0.97 (0.66, 1.41) | 0.854 | **2.53 (1.27, 5.06)** | 0.008 |  |  |
| Quartile 2 | 0.85 (0.58, 1.25) | 0.417 | **2.74 (1.39, 5.39)** | 0.004 |  |  |
| Quartile 1 | 0.86 (0.59, 1.25) | 0.426 | **2.37 (1.21, 4.62)** | 0.012 |  |  |
| *P* for trend |  | 0.344 |  | 0.021 |  |  |
|  | Hypertension (n = 5,204) |  | Non-Hypertension (n = 3,411) |  | 0.023 |  |
| Quartile 4 | Reference | Reference | Reference | Reference |  |  |
| Quartile 3 | 1.37 (0.99, 1.89) | 0.059 | 0.71 (0.40, 1.24) | 0.223 |  |  |
| Quartile 2 | **1.51 (1.10, 2.08)** | 0.011 | 0.58 (0.32, 1.05) | 0.073 |  |  |
| Quartile 1 | **1.56 (1.14, 2.13)** | 0.005 | 0.76 (0.43, 1.33) | 0.331 |  |  |
| *P* for trend |  | 0.007 |  | 0.308 |  |  |
|  | Diabetes (n = 2,090) |  | Non-Diabetes (n = 6,525) |  | 0.747 |  |
| Quartile 4 | Reference | Reference | Reference | Reference |  |  |
| Quartile 3 | 0.94 (0.60, 1.49) | 0.804 | 1.41 (0.98, 2.03) | 0.063 |  |  |
| Quartile 2 | 1.16 (0.75, 1.81) | 0.497 | 1.35 (0.94, 1.94) | 0.108 |  |  |
| Quartile 1 | 1.27 (0.84, 1.92) | 0.264 | **1.45 (1.01, 2.08)** | 0.042 |  |  |
| *P* for trend |  | 0.177 |  | 0.088 |  |  |
|  | Chronic kidney disease (n = 1,115) |  | Nonchronic kidney disease (n = 7,500) |  | 0.208 |  |
| Quartile 4 | Reference | Reference | Reference | Reference |  |  |
| Quartile 3 | 1.20 (0.64, 2.23) | 0.574 | 1.19 (0.86, 1.62) | 0.291 |  |  |
| Quartile 2 | 0.79 (0.43, 1.45) | 0.444 | **1.38 (1.01, 1.89)** | 0.040 |  |  |
| Quartile 1 | 0.95 (0.55, 1.65) | 0.857 | **1.53 (1.12, 2.07)** | 0.007 |  |  |
| *P* for trend |  | 0.566 |  | 0.004 |  |  |
|  | Ever & Current Smoking (n = 4,232) |  | Never Smoking (n = 4,383) |  | 0.660 |  |
| Quartile 4 | Reference | Reference | Reference | Reference |  |  |
| Quartile 3 | 1.29 (0.91, 1.82) | 0.146 | 0.92 (0.56, 1.50) | 0.735 |  |  |
| Quartile 2 | 1.19 (0.85, 1.68) | 0.310 | 1.33 (0.83, 2.13) | 0.233 |  |  |
| Quartile 1 | 1.33 (0.95, 1.86) | 0.099 | **1.57 (1.00, 2.47)** | 0.049 |  |  |
| *P* for trend |  | 0.179 |  | 0.018 |  |  |
|  | Current Drinking (n = 1,866) |  | Noncurrent Drinking (n = 6,749) |  | 0.442 |  |
| Quartile 4 | Reference | Reference | Reference | Reference |  |  |
| Quartile 3 | 1.88 (0.86, 4.09) | 0.112 | 1.06 (0.79, 1.43) | 0.700 |  |  |
| Quartile 2 | 1.38 (0.64, 2.98) | 0.418 | 1.19 (0.88, 1.60) | 0.260 |  |  |
| Quartile 1 | 1.44 (0.68, 3.08) | 0.342 | 1.32 (0.99, 1.76) | 0.062 |  |  |
| *P* for trend |  | 0.686 |  | 0.041 |  |  |
|  | Meet PA Guidelines (n = 2,211) |  | Did Not Meet PA Guidelines (n = 6,404) |  | 0.813 |  |
| Quartile 4 | Reference | Reference | Reference | Reference |  |  |
| Quartile 3 | 1.02 (0.53, 1.97) | 0.949 | 1.20 (0.88, 1.63) | 0.253 |  |  |
| Quartile 2 | 1.30 (0.71, 2.38) | 0.396 | 1.18 (0.87, 1.61) | 0.293 |  |  |
| Quartile 1 | 1.08 (0.58, 2.03) | 0.810 | **1.38 (1.02, 1.86)** | 0.035 |  |  |
| *P* for trend |  | 0.646 |  | 0.047 |  |  |
| PA, physical activity. | | | | |  |  |

| Table S7. Odds ratios (and 95% confidence intervals) for the association of serum α-Klotho concentrations with stroke, stratified by participant characteristics | | | | | |  |
| --- | --- | --- | --- | --- | --- | --- |
|  |  |  |  |  |  |  |
|  | Fully adjusted OR (95% CI) | *P value* | Fully adjusted OR (95% CI) | *P value* | *P* interaction |  |
| Categories | Male (n = 3,967) |  | Female (n = 4,648) |  | 0.604 |  |
| Quartile 4 | Reference | Reference | Reference | Reference |  |  |
| Quartile 3 | 0.83 (0.55, 1.26) | 0.387 | 1.02 (0.70, 1.48) | 0.934 |  |  |
| Quartile 2 | 1.00 (0.67, 1.49) | 0.998 | 0.81 (0.54, 1.20) | 0.289 |  |  |
| Quartile 1 | 0.84 (0.57, 1.26) | 0.404 | 1.06 (0.73, 1.54) | 0.749 |  |  |
| *P* for trend |  | 0.602 |  | 0.982 |  |  |
|  | <60 years old (n = 3,871) |  | ≥60 years old (n = 4,744) |  | 0.013 |  |
| Quartile 4 | Reference | Reference | Reference | Reference |  |  |
| Quartile 3 | 1.07 (0.61, 1.91) | 0.805 | 0.89 (0.65, 1.22) | 0.469 |  |  |
| Quartile 2 | 1.36 (0.78, 2.35) | 0.278 | 0.77 (0.56, 1.07) | 0.117 |  |  |
| Quartile 1 | 1.58 (0.91, 2.71) | 0.101 | 0.79 (0.58, 1.09) | 0.149 |  |  |
| *P* for trend |  | 0.068 |  | 0.115 |  |  |
|  | Non-Hispanic White (n = 4,207) |  | Non-Hispanic Black (n = 1,753) |  | 0.325 |  |
| Quartile 4 | Reference | Reference | Reference | Reference |  |  |
| Quartile 3 | 0.74 (0.48, 1.12) | 0.151 | 1.31 (0.78, 2.18) | 0.305 |  |  |
| Quartile 2 | 0.85 (0.57, 1.28) | 0.445 | 1.11 (0.65, 1.88) | 0.707 |  |  |
| Quartile 1 | 0.73 (0.49, 1.11) | 0.143 | 0.89 (0.52, 1.50) | 0.657 |  |  |
| *P* for trend |  | 0.270 |  | 0.566 |  |  |
|  | Hypertension (n = 5,204) |  | Non-Hypertension (n = 3,411) |  | 0.840 |  |
| Quartile 4 | Reference | Reference | Reference | Reference |  |  |
| Quartile 3 | 0.93 (0.68, 1.26) | 0.621 | 0.94 (0.49, 1.83) | 0.863 |  |  |
| Quartile 2 | 0.89 (0.65, 1.21) | 0.464 | 1.01 (0.52, 1.96) | 0.973 |  |  |
| Quartile 1 | 0.94 (0.70, 1.26) | 0.672 | 1.05 (0.54, 2.05) | 0.880 |  |  |
| *P* for trend |  | 0.671 |  | 0.826 |  |  |
|  | Diabetes (n = 2,090) |  | Non-Diabetes (n = 6,525) |  | 0.714 |  |
| Quartile 4 | Reference | Reference | Reference | Reference |  |  |
| Quartile 3 | 1.07 (0.67, 1.71) | 0.769 | 0.88 (0.62, 1.25) | 0.492 |  |  |
| Quartile 2 | 0.77 (0.48, 1.25) | 0.294 | 0.99 (0.70, 1.40) | 0.960 |  |  |
| Quartile 1 | 1.03 (0.66, 1.59) | 0.910 | 0.94 (0.67, 1.33) | 0.730 |  |  |
| *P* for trend |  | 0.859 |  | 0.922 |  |  |
|  | Chronic kidney disease (n = 1,115) |  | Nonchronic kidney disease (n = 7,500) |  | 0.919 |  |
| Quartile 4 | Reference | Reference | Reference | Reference |  |  |
| Quartile 3 | 0.90 (0.47, 1.72) | 0.755 | 0.95 (0.69, 1.29) | 0.728 |  |  |
| Quartile 2 | 0.96 (0.53, 1.75) | 0.903 | 0.92 (0.67, 1.26) | 0.609 |  |  |
| Quartile 1 | 0.88 (0.50, 1.53) | 0.638 | 1.05 (0.77, 1.43) | 0.755 |  |  |
| *P* for trend |  | 0.683 |  | 0.797 |  |  |
|  | Ever & Current Smoking (n = 4,232) |  | Never Smoking (n = 4,383) |  | 0.254 |  |
| Quartile 4 | Reference | Reference | Reference | Reference |  |  |
| Quartile 3 | 0.94 (0.65, 1.36) | 0.751 | 0.92 (0.60, 1.41) | 0.706 |  |  |
| Quartile 2 | 0.94 (0.65, 1.35) | 0.731 | 0.91 (0.59, 1.41) | 0.664 |  |  |
| Quartile 1 | 1.06 (0.74, 1.50) | 0.755 | 0.88 (0.58, 1.34) | 0.547 |  |  |
| *P* for trend |  | 0.703 |  | 0.554 |  |  |
|  | Current Drinking (n = 1,866) |  | Noncurrent Drinking (n = 6,749) |  | 0.034 |  |
| Quartile 4 | Reference | Reference | Reference | Reference |  |  |
| Quartile 3 | 1.33 (0.58, 3.09) | 0.499 | 0.89 (0.66, 1.19) | 0.434 |  |  |
| Quartile 2 | 0.97 (0.42, 2.25) | 0.948 | 0.89 (0.66, 1.20) | 0.449 |  |  |
| Quartile 1 | 0.57 (0.23, 1.38) | 0.213 | 1.04 (0.78, 1.38) | 0.809 |  |  |
| *P* for trend |  | 0.096 |  | 0.748 |  |  |
|  | Meet PA Guidelines (n = 2,211) |  | Did Not Meet PA Guidelines (n = 6,404) |  | 0.581 |  |
| Quartile 4 | Reference | Reference | Reference | Reference |  |  |
| Quartile 3 | 1.53 (0.72, 3.27) | 0.272 | 0.88 (0.65, 1.19) | 0.400 |  |  |
| Quartile 2 | 1.21 (0.57, 2.58) | 0.614 | 0.87 (0.65, 1.18) | 0.372 |  |  |
| Quartile 1 | 1.31 (0.61, 2.82) | 0.484 | 0.91 (0.68, 1.21) | 0.518 |  |  |
| *P* for trend |  | 0.683 |  | 0.570 |  |  |
| PA, physical activity. | | | | |  |  |
